# Drone-Based Statistical Detection of Methane Anomalies Around Abandoned Oil and Gas Well Sites

**DOI:** 10.3390/s26072205

**Published:** 2026-04-02

**Authors:** William Hoyt Thomas, Caixia Wang

**Affiliations:** GeoComputing Laboratory, Department of Geomatics, University of Alaska Anchorage, Anchorage, AK 99508, USA; whthomas97@gmail.com

**Keywords:** abandoned wells, methane, drones, oil and gas, laser gas detector

## Abstract

Abandoned oil and gas wells pose significant risks to human health and the environment by emitting air pollutants, contaminating groundwater, and leaving behind hazardous debris. In the United States, approximately 3.9 million documented wells vary widely in the accuracy of their recorded locations and plugging status, creating major challenges for detection, mapping, and remediation. Existing well detection methods show some promise but often lose effectiveness under complex conditions, such as vegetation occlusion or construction without metal components. In this study, we propose a drone-based approach equipped with a highly sensitive methane sensor to identify statistical anomalies in methane concentrations around abandoned oil and gas well sites. To address the noisy and variable nature of environmental sensor data, statistical methods were developed that enable reliable anomaly detection under field conditions. Controlled release experiments with known emission points validated the method’s ability to statistically detect methane anomalies that may indicate nearby emission sources. We further tested the approach at a field site containing three abandoned wells with known locations and sparse emission profiles. The results demonstrate that the proposed drone-based sensing method can serve as a rapid survey approach to identify areas with elevated methane signals around well sites, helping to reduce the scope of the ground survey area, and supporting prioritization of follow-up ground investigations. This approach provides a practical means to support targeted monitoring and prioritization of remediation efforts, while supporting the future development of source attribution and localization methods.

## 1. Introduction

The oil and gas (O&G) industry has operated in the US for over 200 years, yet not until the Clean Air Act of 1963 did environmental protection begin to be legislated and enforced. According to the United States Environmental Protection Agency’s report on the Inventory of Greenhouse Gas Emissions and Sinks in 2024, there are about 3 million abandoned wells nationwide [1]. An ‘abandoned’ well is a well no longer in production, has a legally responsible owner and can be either plugged or unplugged. In addition to abandoned wells, ‘orphaned’ wells are a category of unplugged, nonproducing wells with no legally responsible party [2]. In 2023, the number of documented orphaned wells was 117,672 [3] and the number of undocumented orphaned wells was estimated between 310,000–800,000 [4]. In this work, both abandoned and orphaned O&G wells are collectively referred to as abandoned O&G wells, recognizing that terminology may vary by state.

Research in the last 10 years has shown unplugged, abandoned O&G wells constitute a consistent pathway for Methane (CH_4_) to be fugitively released to the atmosphere and damage environmental/human health [5,6,7,8,9]. Methane emissions from abandoned wells can occur when well integrity degrades and creates pathways for gas migration from subsurface formations to the surface. Potential pathways include leakage along the wellbore, migration through annular spaces between casing and surrounding formations, or transport through compromised cement or plugging materials. Methane may originate from residual thermogenic hydrocarbons in previously producing reservoirs or from microbial methanogenesis in shallow formations. The occurrence and magnitude of emissions can vary substantially depending on factors such as well type, plugging status, well age, construction practices, and regional geology [10,11,12]. CH_4_ is a prominent greenhouse gas with a short atmospheric lifespan of ~10 years. Compared to carbon dioxide (CO_2_), another greenhouse gas, the global warming potential of CH_4_ over 20 years is 86 times greater. Due to its properties, CH_4_ has been targeted by many climate change mitigation initiatives as benefits from reductions would be realized sooner than would be from reducing CO_2_. CH_4_ is emitted naturally as a part of the carbon cycle and anthropogenically from three main industries: Oil and Gas (O&G), Solid Waste Management, and Agriculture. Among these anthropogenic sources, the US Oil & Gas (O&G) industry accounts for ~30–40% of its country’s CH_4_ emissions [13]. The US EPA estimates a quantity of CH_4_ of 282 million tons of CO_2_ equivalent with abandoned wells contributing 8.5 million tons of CO_2_ equivalent or about 3% of the sector’s total CH_4_ emissions [1]. It is worth noting the considerable uncertainty present in these estimates: Relative to millions of abandoned wells, only 1125 wells, less than 0.03% of total abandoned wells, have been directly measured [14]. In addition to the climate risk from CH_4_ emissions, abandoned wells present risks to human and environmental health. Unplugged wells provide a pathway for remaining oil or gas to migrate to the surface and contaminate ground and surface water resources. Benzene, a known carcinogen, can be expected to be co-emitted with CH_4_; one study of wells in Pennsylvania detected benzene at 70% of sites. The buildup of CH_4_ in an enclosed space can lead to dangerous explosions when the lower explosive limit is reached [15]. When left improperly remediated, plugged or unplugged wells also pose a risk to areas where the land use has changed. For example, Oil Creek State Park, Pennsylvania, is an area where abandoned wells have been studied and is now open to the public as a recreation area [16]. Deteriorated O&G infrastructure could injure those visiting the area.

With the risks documented, Section 40601, Title IV of the 2021 Bipartisan Infrastructure Law (BIL) made $4.675 billion of federal funds available for plugging, remediation, and restoration of orphaned well sites. The majority ($4.275 billion) of these funds were for the 28 states with documented abandoned wells, for which the respective states are responsible. The number of documented abandoned wells in each state determines the portion of funds received by state agencies. In Alaska, twelve documented abandoned wells have equated to $28,346,487 received in two phases of grants, the first in 2024 and the second in 2025 [17]. However, among the combined 3.9 million abandoned wells, characteristics vary, as do their meaningful effects on environmental risks and remediation strategies. Well age, integrity, depth, and plugging status have all been found to be correlated with greater CH_4_ emissions [11].

Methods to locate abandoned wells have largely been limited to aerial magnetometry surveys [6,13,18,19]. By detecting the magnetic anomalies created by steel well casings, these surveys have demonstrated the capabilities of the sensor but noted shortcomings related to measurement noise [19] and false positives caused by background magnetic anomalies [16]. A handheld Laser CH_4_ sensor using Tunable Diode Laser Absorption Spectroscopy (TDLAS) was used to verify leaks from abandoned wells on the ground in a prior work [8], and the technology has been used by the O&G and solid waste industry for leak detection and repair (LDAR) for at least 20 years [20]. Recent studies, coinciding with the proliferation of drone usage, have studied the use of drone mounted TDLAS sensors to detect CH_4_ emissions in landfill, permafrost, and gas pipeline contexts [21,22,23]. Another study of a TDLAS-drone combo system tested the system at Methane Emissions Technology Evaluation Center (METEC), a CH_4_ detection technology testing center operated by Colorado State University. Surveys showed that measurements of natural gas (~85% CH4) from wellheads, tanks, and separators follow an approximately normal distribution in the absence of leaks, but become skewed when a methane leak is present. A skewness threshold separated the leak from no-leak scenarios with zero false positives or false negatives [24].

Building on these advances, this work investigates the use of drone-mounted methane sensing combined with statistical analysis to detect methane anomalies around abandoned oil and gas well sites. Rather than directly identifying wells or performing source localization, the objective is to characterize anomalous methane patterns that may indicate the presence of nearby emission sources, thereby supporting rapid field surveys and prioritization of follow-up investigations.

## 2. Materials and Methods

### 2.1. Methane Collection System

**Figure 1:**
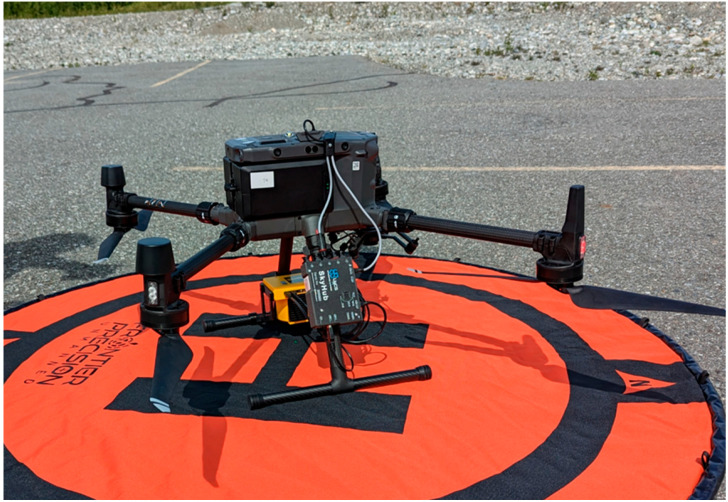
Hardware components of the methane survey system, excluding the UgCS software v4.x.

The methane survey system comprised a Matrice 300 RTK (M300 RTK) drone platform, a statically mounted CH_4_ detection sensor, a lightweight onboard computer, and a specialized UgCS software (Figure 1). The M300 RTK is a quadrotor drone powered by two 12S lithium polymer (LiPo) batteries which provide up to 55 min of flight time without payload. An UgCS Skyhub served as the onboard computer to communicate between the drone, methane sensor, and the UgCS software from the ground control station. It also logged data, synchronized GNSS coordinates of drone locations with CH_4_ measurements, uploaded flight routes, and allowed direct drone control via UgCS software.

CH_4_ detection was performed by using a Pergam Laser Falcon, a tunable diode-laser absorption spectroscopy (TDLAS) CH_4_ sensor capable of measuring CH_4_ column density within a range of 0–50,000 parts-per-million-per-meter (ppm-m) with an accuracy of ±10% of the measured value. This high CH_4_ sensitivity is crucial to the proposed methodology, as methane emissions in the field are typically sparse and intermittent. The Laser Falcon emits infrared laser light at a wavelength of 1.653 µm, corresponding to a strong absorption line of CH_4_ [23]. As the laser beam travels to the ground surface and back to the sensor, methane along the optical path absorbs part of the radiation at this wavelength. The sensor measures the attenuation of the returned signal and converts it to a path-integrated methane concentration along the laser beam. The internal reference cell provides a built-in calibration check, and a self-calibration protocol was initiated prior to each measurement. Details of the underlying physical mechanism can be referred to the work of [25]. Because this study focuses on identifying methane anomalies relative to nearby observations rather than quantifying absolute methane concentrations, measurements are interpreted by comparing local enhancements with neighboring measurements obtained under the same survey conditions. Potential influences such as plume geometry, background reflectivity, and atmospheric scattering may affect the returned signal but are mitigated through the relative anomaly-based detection approach and the instrument’s internal calibration. The Laser Falcon was powered by an independent three-cell 11.1 V LiPo battery. With the added payload, the M300 RTK’s flight time was reduced to approximately 35 min, and variable wind conditions could further shorten operational duration.

For each field site, a pre-mission site visit was conducted to assess the area, determine a safe flight altitude, and identify potential obstacles for the drone. Because the methane surveys benefit from flying at the lowest possible altitude to maximize the likelihood of detecting emissions before they disperse, careful measurement of obstacle heights is critical for flight safety. A rangefinder, commonly used in forestry, utility, and construction, was employed for this purpose.

The collected information was used to define the flight boundaries and create mission plans for grid-based area scans. The spacing between flight passes and the selected flight speed were determined based on the sensor’s field of view at a given altitude and its data acquisition rate. These parameters ensured approximately one CH_4_ measurement every meter and provided a sufficient dense grid to cover the area of interest. Whenever possible, the best practice is to design flight areas to be completed within a single flight. However, the UgCS software enabled route interruptions for battery replacement and can resume the mission from a designated waypoint if needed.

On-site surveys began with additional safety checks on obstacle heights and stability of attached payloads. Prior to take-off, wind speed and direction were recorded to document environmental conditions and provide contextual information for the survey. These measurements were taken before completing a pre-mission safety checklist and were not incorporated in the subsequent data analysis. Due to the attached payloads, downward-facing obstacle avoidance sensors were deactivated. The M300 RTK has capability to automatically take-off and land but without downward sensors, it would use positioning based on GNSS locating and publicly available elevation models. To ensure safety of equipment and operators, manual take-off, taxiing, and landing are preferred when using the system. Once in the air, a controller responsiveness check was completed and then the survey could begin. Following the manual landing, collected data was downloaded off the on-board computer to begin the analysis process.

### 2.2. Pre-Processing

Laser path length during surveys with a TDLAS sensor is an important consideration when processing data as CH_4_ measures are collected as ppm-m. Previous work with TDLAS sensors to detect CH_4_ [23] found that potential laser path length variations caused by non-linear drone movements and unaccounted for ground variations are main sources of uncertainty when using these sensors. For statically mounted sensors, an unexpected roll, tilt, or yaw moves the sensor off nadir-view, thus increasing laser path length, and potentially result in a false detection. If the background CH_4_ levels are significant, an increase in the path length will include more background CH_4_ in the recorded measure. As such, data preprocessing is necessary to remove measurements collected under conditions where path lengths are likely to change. The primary scenarios identified include takeoff, taxiing, landing, and turns. As the drone adjusts its velocity when entering, exiting or executing turns, the sensor may be displaced from its nadir-view position. To minimize the impact of these deviations, data collected during taxiing, takeoff, and landing were excluded using timestamps aligned with the recorded start and end times of each flight. Similarly, during stops and turns between flight paths, the drone slows down to complete the maneuver; therefore, a velocity threshold was identified and applied to filter out measurements obtained during the turns.

### 2.3. Skewness Analysis

Skewness serves as an effective statistical measure for distinguishing leak from no-leak scenarios based on the probability density distribution of CH_4_ measurements [24]. In no-leak scenarios, histograms of CH_4_ measurements tend towards a normal distribution, reflecting background methane levels with symmetric variation. In contrast, leak scenarios exhibit positive skewness, characterized by a long right tail in the distribution

This statistical measure is incorporated into the proposed methodology. The unbiased skewness of each histogram is calculated following Equation (1) provided by [26]:

(1) $$g_{1}=\frac{\frac{1}{N}\sum_{n=1}^{N}{\left(x_{n}-\bar{x}\right)}^{3}}{{[\frac{1}{N}\sum_{n=1}^{N}{\left(x_{n}-\bar{x}\right)}^{2}]}^{3/2}}.$$

A skewness threshold is then determined experimentally to distinguish between leak and no-leak scenarios.

### 2.4. Clustering Analysis

Although skewness shows promise for characterizing the data distribution, it is crucial to determine an appropriate threshold to distinguish leak from non-leak cases. This challenge arises because methane can occur naturally in the environment due to factors such as nearby wetlands, landfills, or similar conditions that emit methane into the atmosphere. To address this, we apply spatial autocorrelation analysis to further characterize leak cases, which are expected to exhibit spatial patterns that are statistically distinct from those of non-leak cases. Spatial autocorrelation quantifies the degree to which values observed at nearby locations are similar or dissimilar, providing a useful measure for identifying spatial patterns. When nearby locations exhibit similar values, positive spatial autocorrelation is present, and the distribution is characterized by the clustering of similar values; conversely, negative spatial autocorrelation indicates that nearby locations tend to have dissimilar values [27]. Among the various measures of spatial autocorrelation, Moran’s I and Geary’s C are the most widely used, while Moran’s I is generally preferred due to its higher sensitivity, ease of interpretation, and consistency with other spatial statistics [27,28,29]. In this study, Global Moran’s I statistic, which provides a single summary value representing the overall degree of spatial autocorrelation, was employed. This measure is suitable for CH_4_ leak detection, as leak events are expected to produce spatially clustered high CH_4_ concentrations that differ significantly from the more dispersed background levels associated with nature sources such as wetlands, or landfills. Global Moran’s I is calculated using Equation (2) [30]:

(2) $$I=\frac{N}{S_{0}}\frac{\sum_{i=1}^{N}\sum_{j=1}^{N}w_{i,j}z_{i}z_{j}}{\sum_{i=1}^{N}z_{i}^{2}},$$

where *z_i_* is the deviation of the value measure *i* from its mean (*x_i_* − $\bar{x}$), *w_i,j_* is the spatial weight between feature *i* and *j*, *N* is equal to the total number of features, and *S*_0_ is the aggregate of all the spatial weights.

**Figure 2:**
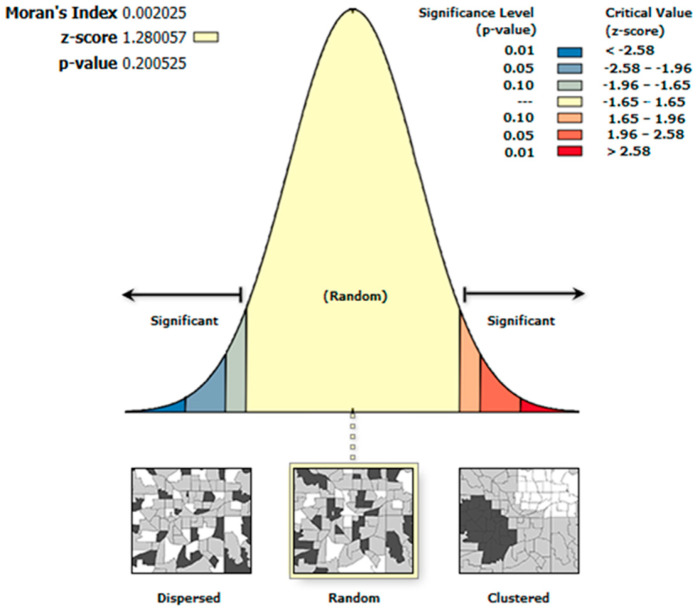
Normal Distribution with varied confidence thresholds.

As with most statistical tests, the Moran’s I test begins with the formulation of a null hypothesis. For spatial pattern analysis, this null hypothesis is Complete Spatial Randomness (CSR), which assumes that the observed spatial distribution of values is random and exhibits no spatial autocorrelation. In the context of methane detection, CSR implies that methane concentrations are spatially independent, with no clustering attributable to leak events. Along with the calculated Moran’s I, the test returns a z-score and a *p*-value, which indicates whether the null hypothesis can be rejected. The z-score represents the standardized value of the observed statistic relative to its expected value under CSR, expressed in terms of standard deviation. It is calculated as:

(3) $$Z=\frac{I-E[I]}{\sqrt{V[I]}},$$

where *E*[*I*] is the expected normal distribution of *I* and *V*[*I*] is the variance of *I*. The *p*-value represents the probability that the observed spatial pattern could have arisen from a random process. A very small *p*-value indicates that such a pattern is not highly likely to result from randomness, allowing us to reject the null hypothesis [31]. In leak cases, which are characterized by positive Moran’s I value, we expect the corresponding z-score and *p*-value to reveal statistically significant clustering of methane measurements, rather than a random spatial distribution. Accordingly, we reject the null hypothesis when these metrics indicate non-randomness. The z-scores and *p*-values used in this work are interpreted in accordance with the standard normal distributions (Figure 2).

The scale of spatial pattern analysis plays a critical role in Moran’s I calculation because it determines the neighborhood over which spatial relationships are evaluated. Mathematically, the scale affects the spatial weights matrix, which is used to calculate *S_0_* and the corresponding Moran’s I values for each location. Two key parameters must be defined: the neighborhood size (spatial extent) and the mathematical weighting of observations within that neighborhood. An incremental spatial autocorrelation analysis was performed to determine the neighborhood size by identifying the distances at which clustering was most significant in the data set. This was achieved by calculating *I* according to Equation (2) at the minimum neighborhood distance, where a point only has one neighbor, and then increasing the neighborhood distance incrementally [32]. The result of the process is a plot comparing clustering significance and distance. From this plot, a peak may be identified, which can be interpreted as the distance at which clustering is most pronounced [33]. In the context of methane leak detection, this can be interpreted as the approximate size of a CH_4_ plume, that is, the distance over which elevated methane concentrations are spatially correlated.

The weighing scheme specifies how the influence of one location’s value diminishes with distance from another. Common approaches include:

- Inverse Distance (ID): Weight = 1/*d*, where *d* is the distance between locations.
- Inverse Distance Squared (ID^2^): weight = 1/*d*^2^, giving strong emphasis to nearer neighbors.
- Fixed Distance (FD): weight = 1 for all locations within the neighborhood.
- Zone of Indifference (ZoI): Weight = 1 within the neighborhood and 1/*d* beyond it.

These parameter choices directly affect the sensitivity of Moran’s I to spatial patterns, and the optimal configuration should be determined experimentally for the specific application.

### 2.5. Statistical Identification of Methane Anomalies

To distinguish methane anomalies from background variability, the two statistical indicators introduced previously, skewness and Global Moran’s I, were combined to assess their consistency. As described earlier, skewness characterizes the asymmetry of methane signal distributions obtained during each flight survey. Under relatively uniform conditions and constant flight altitude, methane measurements are expected to be roughly normally distributed around the background level. The presence of a nearby methane source can introduce a small number of elevated observations, resulting in a positively skewed distribution. Global Moran’s I, described in the preceding section, was used to determine whether these elevated observations exhibit spatial structure rather than random variability by assessing spatial autocorrelation among methane measurements. Positive Moran’s I values indicate spatial clustering of similar values, whereas values near zero indicate a random spatial distribution. The statistical significance of the spatial autocorrelation was evaluated using the associated *p*-value; a small *p*-value indicates the observed clustering is unlikely to arise from a random process. The joint evaluation of skewness and Moran’s I, therefore, provides a statistical basis for distinguishing spatially coherent methane anomalies from isolated observations resulting from background fluctuations or sensor noise.

## 3. Results and Discussion

### 3.1. Controlled Release

A series of controlled release tests were performed at a designated test site to validate the proposed methane detection methodology using the Methane Survey System. These tests were carried out at the University of Alaska Anchorage (UAA) Mat-Su campus in Palmer, Alaska, hereafter referred as Mat-Su site. This location was chosen due to its class G airspace classification and a large, unoccupied, and leveled lot on the north side of the property, which provided a safe and controlled environment for repeated drone flights.

To simulate a leaking well, methane was released at a constant rate of 0.8 L per minute (LPM) using a 5% CH_4_ calibration gas mixture. The exact release location was recorded to compare with the detection results. Four flights (Tests A–D) were conducted: one flight without methane release (no-leak case) and three flights with the simulated leak (leak cases). Each flight flew the same approximate 44 m × 47 m grid pattern, with 2 m spacing between flight paths and a flight speed of 2 m/s. Flights were conducted at varying altitudes to assess the impact of altitude on methane detection performance [22]. The no-leak case control flight was conducted at 32 m above ground level (AGL), while the three simulated leak flights were flown at altitudes of 17 m AGL, 20 m AGL, and 25 m AGL, respectively.

Preprocessing was applied to the raw measurement to remove erroneous measures associated with taxiing, takeoff, and landing by using timestamps aligned with the recorded survey start and end times. Furthermore, turning maneuvers were removed by filtering out all measurements below a velocity threshold of 1.8 m/s.

We implemented the proposed skewness analysis on the filtered CH_4_ measurements. Results showed surveys with a leak present have a noticeably higher skew. Test A with no leak present has a skewness of 1.41 compared to an average skewness of 9.61 from Tests B–D (Table 1).

Skewness results from the controlled release tests indicate a threshold of 1.5 is effective for distinguishing leak cases. This finding aligns well with the previously established threshold [24]. However, a higher threshold may be needed in field cases where either the laser path length is more variable or elevated background CH_4_ levels are present. A more variable path length could lead to step changes in ppm-m measures, which push skew higher. Elevated background CH_4_ levels similarly affect skew if a CH_4_ source previously unaccounted for has spread into the test area. Flight altitude could also affect skew, as shown in the controlled release tests. With a leak present, a greater proportion of the CH4 plume could be observed by a longer laser path length, possibly resulting in higher CH_4_ readings and a higher skew, whereas, with no leak present, the increased laser path length could dampen any fluctuations in path length due to ground conditions. As this relationship is influenced by atmospheric dynamics and stability is not quantified in this study, the previously established threshold of 1.5 from the work of Golston et al. [24] is used.

**Figure 3:**
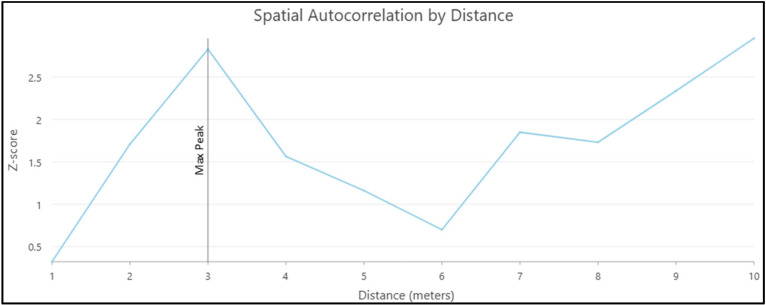
Incremental Spatial Autocorrelation graph from Test B with max peak indicated at 3 m.

**Figure 4:**
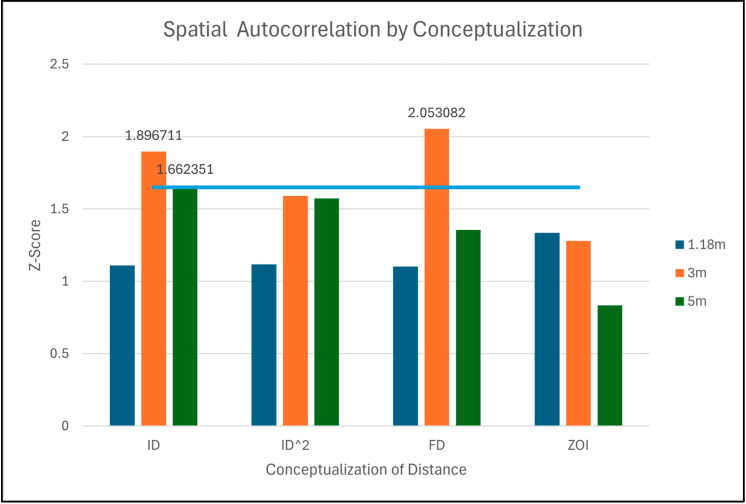
Comparison of Conceptualization of Distance methods used to calculate Moran’s I. Grouped by method and colored by distance band with a threshold of statistical significance (shown as a solid blue line). Z-Scores of those methods surpassing the threshold have their values labeled.

Global Moran’s I clustering analysis is sensitive to both neighborhood size and method of conceptualizing the distance weights between points within a neighborhood. Incremental Spatial Autocorrelation (ISA) was used to determine the appropriate neighborhood size. ISA finds the peak z-scores, which indicate high spatial autocorrelation at the corresponding neighborhood sizes (distances) (Figure 3). During ISA, the first peak is used because it often best reflects the scale of the question, and later peaks may represent spatial patterns beyond the scale of the test set [31]. Using Test A point data, a comparison of the conceptualization of distance methods was completed by calculating Global Moran’s I for each method at three different neighborhood distances. Distances of 1.18, 3, and 5 m were selected, as they were the first three peaks from the ISA graph. The findings indicated ‘Zone of Indifference,’ as the conceptualization of distance exhibits the most stable results across all cases for clustering analysis (Figure 4).

Results from Moran’s I indicate Tests B–D with a statistically significant clustered pattern and an average z-score of 4.78, corresponding to a less than 1% chance the spatial clustered pattern was generated randomly (Table 1). Conversely, Test A without a leak present showed a pattern not significantly different from a random pattern. This statistical measure further validates the findings from the skewness analysis. The agreement between Global Moran’s I and skew results indicates using both could act as a two-step verification process. However, the max skew value does not align with the max z-score, demonstrating the sensitivity of each method. As skew does not consider the spatial distribution of measures, it is more sensitive to extreme outliers regardless of where they appear in relation to neighboring measures. Whereas Moran’s I consider outliers in relation to the nearby measures, the neighborhood is described by a distance band and a conceptualization of distance, and is more sensitive to clustering of higher measures. If a survey has a low skew, Global Moran’s I could still indicate significant clustering if the relatively ‘high’ measures from the survey are near each other. This scenario would likely be indicative of a first-order effect at test and demonstrate that methods should be used in conjunction to inform if a true leak case is present.

### 3.2. Field Site Verification

**Figure 5:**
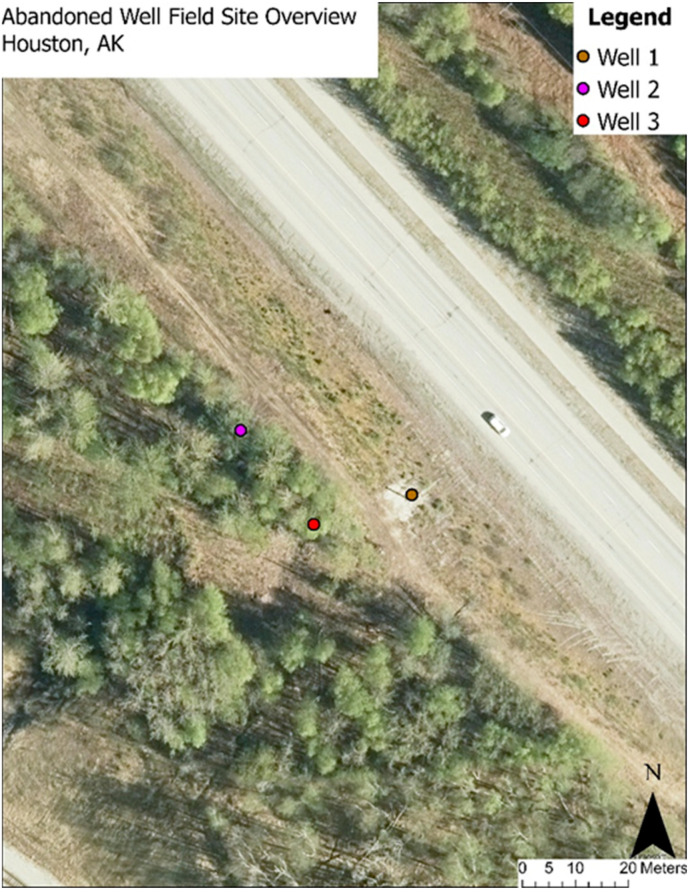
Field Site Overview.

**Figure 6:**
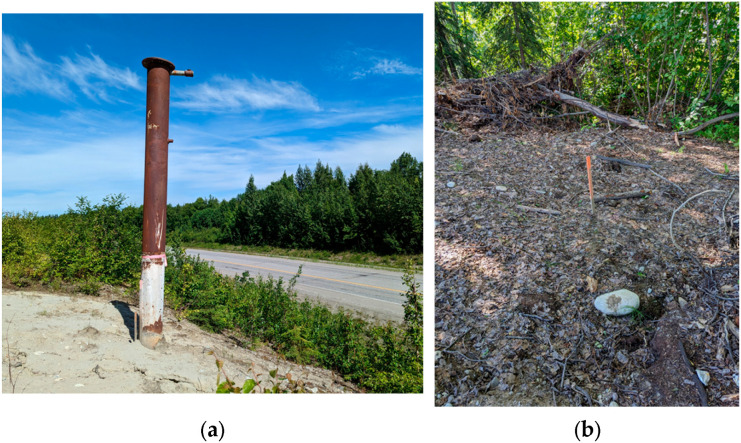
(**a**) Well 1 located on bare ground; (**b**) Well 2 surrounded by trees.

Three wells, located in Houston, Alaska, were selected for this study using available regulatory resources They were identified in close proximity (Figure 5), enabling a single field survey site and allowing for in-situ verification of the proposed methodology. Additionally, according to the progress summary report conducted by an engineering consulting firm, Well 1 (Figure 6a) was previously plugged, rose approximately 2 m above the ground, and is located closest to the highway. CH_4_ measurements taken by the engineering firm from a photoionization detector (PID) indicated low levels of gas from outside the well casing, though exact leak rates were not provided. Well 2 (Figure 6b) is unplugged and in the wooded area, buried approximately 0.5 m below ground, and was excavated to take PID measurements. These measures indicated active leaking from the well casing, and water was also leaking from the casing. However, the exact leak rate was also not provided, and the well itself was re-buried prior to this study. Well 3, also in the wooded area, was initially thought to be a water well and was excavated to identify it as an unplugged gas well. PID measures also indicated the well was leaking gas, though with no exact figure provided. Therefore, a no-leak case was not available for the field tests.

The CH_4_-based detection methodology was tested in the field study area previously described. CH_4_ data were collected across three Surveys (1–3): Surveys 1 and 2 conducted before remediation and Survey 3 during the remediation process. Each survey consists of two flights: one at 25 m AGL covering all three wells and another at a lower altitude of 20 m AGL focusing on Well 1, enabled by the absence of obstacles at the site. For example, Survey 1 included Flights E and F, where Flight E has 25 m AGL, covering Wells 1, 2, and 3, and Flight F has 20 m AGL, focusing on Well 1.

Additionally, the 25 m AGL survey data that covers all three wells were divided to reflect ground conditions: Wells 2 and 3 were located beneath tree cover, while Well 1 was situated in open ground (Figure 5). This division helped account for potential differences in methane dispersion due to canopy interference. However, the 25 m AGL survey conducted during remediations (Survey 3) was not divided, as the site had been leveled and all vegetation obscuring the wells had been cleared (Table 2).

**Figure 7:**
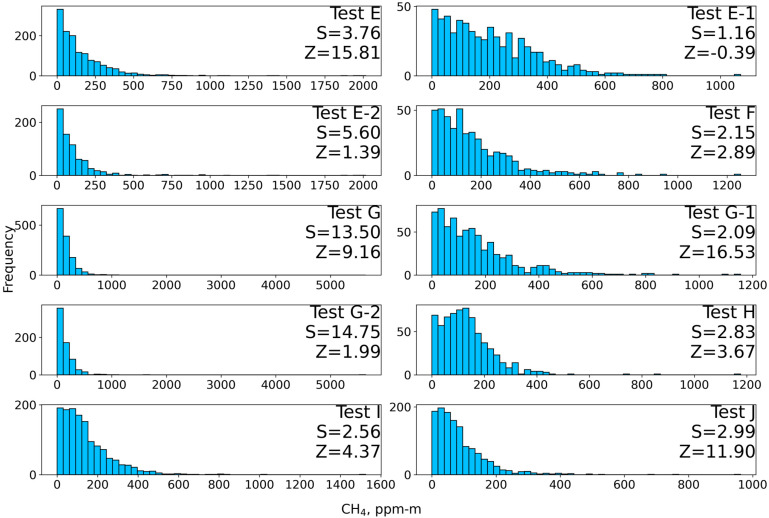
Test E–H histograms of filtered ppm-m measures with test name, skewness (S), and Z-score (Z) from Global Moran’s I superimposed. Note: The histogram axes are scaled individually to better illustrate the distribution characteristics and long-tailed behavior of the measurements.

In field surveys, the intended survey area may not always be covered as one spatially continuous flight segment because of local obstacles and tree height. In such cases, the survey area is divided into subsets similar to E-1 and E-2, and skewness and Moran’s I were computed for each subset individually (Figure 7). Because both statistics depend on the measurements included in the analysis, the values obtained for these subsets are not intended to be directly equivalent to those from a fully contiguous survey of the complete area. Instead, they are used to evaluate the proposed methane enhancement detection framework under realistic field conditions, where only partial spatial coverage may be achievable.

Tests E and G covering Wells 1–3 both have skewness above the threshold of 1.5 and have z-scores indicating less than 1% likelihood the spatially clustered pattern is the result of random chance. The division data from Tests E and G also indicate there is a CH_4_ leak present; the results disagree over whether the leak is from Well 1 (Tests E-1, G-1) or Wells 2 and 3 (Tests E-2, G-2). Tests E-1 and E-2’s skewness, 1.16 and 5.60, respectively, indicate higher measurements originate from Wells 2 and 3, but neither test indicates statistically significant clustering. The z-score for Test E-1 is negative but small, indicating there is no statistically significant pattern present. While E-2’s z-score is under the 1.65 threshold, a 1.39 z-score is still associated with only a 16% likelihood a pattern is the result of random chance. Tests G-1 and G-2’s skewness, 2.09 and 14.75, respectively, also indicate higher measurements originate from Wells 2 and 3, and both are above the 1.5 skewness threshold. Both Tests G-1 and G-2 also indicate statistically significant clustering. The likelihood of clustering resulting from random chance is much lower for G-1 than G-2, indicated by their z-scores of 16.53 and 1.99. Tests F and H indicate Well 1 may be potentially leaking or methane was being transported from nearby emission sources during the survey, with skewness of both tests above the 1.5 threshold, 2.15 and 2.83, respectively. Their z-scores indicate statistically significant clustering in both tests, 2.89 and 3.67, respectively.

Tests I and J indicate a leaking well is still present at the time of this survey. Skewness values from both tests are above the 1.5 threshold and z-scores from Moran’s I indicate statistically significant clustering.

In summary, the skewness values indicate at least one well in the study area was emitting CH_4_ during all survey periods, both before and during remediation. The spatial clustering detected is statistically significant, further suggesting the presence of one or more wells of interest within the study area. Methane emissions in the area prior to remediation were further confirmed through site visits by the engineering firm.

### 3.3. Field Site Discussion

Field experiments demonstrate the effectiveness of the proposed methodology in identifying leak cases, which was further validated through third-party site visits. However, several important considerations should be pointed out. The skewness threshold of 1.5 was surpassed in every test except E-1. Z-scores from Moran’s I indicate statistically significant clustering in all but two surveys, E-1 and E-2. Notably, E-2 was only 6% below the 90% confidence threshold for clustering. As with the skewness results, the methane distribution in Test E-1 appeared random, which was assumed to be due to dynamic wind interference, as with previous studies [24]. Taken together, these findings strongly suggest there is at least one well leaking CH_4_ at the site, even during the remediation process. However, the disagreement between some z-scores and skewness values highlights issues previously discussed, particularly the influence of wind conditions. As the site was located next to a busy highway, intermittent gusts from passing cars would occasionally overpower the prevailing wind pattern measured prior to flight.

Although some portions of the field survey exhibited relatively lower methane variability, the site was known to contain methane leaks from multiple wells based on information provided by the engineering consulting firm. Because methane emissions and plume transport may influence nearby measurements, we did not designate specific regions within the survey as background conditions. Future studies at sites where emission-free areas can be confidently identified may benefit from incorporating background-region analyses to provide additional context for interpreting spatial statistics.

The effects of laser path length are also evident across the three surveys. Tests E and F took place after spring green-up had already occurred, Tests G and H took place after fall, and Tests I and J took place after remediation work had cleared all vegetation cover and exposed the previously buried Wells 2 and 3. Compared to the skewness of the full area Tests E, skewness values of Tests E-1 and G-1 decreased when isolating the unvegetated area around Well 1 (thus, a longer and more consistent laser path length), and increased for Tests E-2 and G-2 when isolating the vegetated area around Wells 2 and 3 (thus, a variable and shorter laser path length). Despite those challenges, the skewness results are consistent with prior knowledge that Wells 2 and 3 were unplugged and leaking greater quantities of CH_4_. The effect of laser path length is more evident in the z-scores as inconsistencies in path length are more likely to reduce the number of possible elevated measures in a neighborhood. From summer to fall tests, z-scores increased in both divisions of the surveys (E and G). However, results from Tests E, E-1, and E-2 indicated dividing a completed survey in post-processing may remove neighborhood context important to the Moran’s I algorithm, evidenced by the z-score from Test E decreasing to below significance levels in Tests E-1 and E-2. Z-scores indicating statistical significance from the Tests I and J following the removal of vegetation also highlight the importance of a consistent laser path length for this methodology.

## 4. Conclusions and Future Work

The combined skewness and clustering analysis was successful at detecting methane anomaly patterns both at the control and field sites. The results from both the control and field sites provide evidence supporting the effectiveness of the statistical indicators used in this study. Measurements collected at the control site for no-leak cases were approximately symmetric around the background level, with skewness values close to zero and Global Moran’s I values near zero, indicating no significant spatial clustering and consistency with background variability. In contrast, measurements collected over field sites with suspected methane emissions showed positively skewed distributions and significantly positive Moran’s I values with small *p*-values. These results indicate that elevated methane observations occur in spatially clustered patterns rather than as isolated measurements or random fluctuations. The combined evaluation of skewness and Global Moran’s I therefore provides a basis for distinguishing spatially coherent methane anomalies from background variability or sensor noise in the survey data.

Despite the encountered challenges related to atmospheric conditions and sensor laser path length, the statistical thresholds used are consistent with prior work, were confirmed using the controlled CH_4_ release, and demonstrate robustness for detecting methane anomalies in field survey conditions. This methodology can be applied to additional CH_4_ LDAR tasks to distinguish when surveys contain anomalous CH_4_ observations when a TDLAS CH_4_ sensor is used. Regardless of the task, future surveys should design flight plans around key considerations discussed and confirm the presence and origin of anomalous methane signals with a ground-level investigation.

This study demonstrates the ability to detect statistically significant methane anomalies around abandoned oil and gas well sites using drone-based sensing and statistical analysis. The proposed framework is intended as a rapid survey approach to identify areas exhibiting elevated and spatially clustered methane signals, which can inform prioritization of follow-up ground investigations. While precise source attribution is beyond the scope of this work, the anomaly detection framework established here provides a foundation for integrating atmospheric transport modeling and inversion methods in future studies.

Future work should also explore methods to detect abandoned wells that are not emitting CH_4_. For this subset of wells, methods could take advantage of additional well or well-site characteristics. Such methods could also leverage deep learning techniques to lessen the overall amount of direct surveying, which would be done using traditional methods. While there has been recent work applying Deep Learning based frameworks using Convolutional Neural Networks (CNNs) as backbones to detect O&G infrastructure [34,35,36,37,38], few have been in the application of identifying abandoned oil and gas wells. For these frameworks to be successful, input data needs to be resilient to surface-level obstructions associated with well site deterioration, vegetation overgrowth, and varying terrain conditions.

## Figures and Tables

**Table 1:** Summary of results of controlled methane release Tests A–D. Each test covered approximately the same area of 44 m by 47 m.

Test	Flight AGL [m]	Number of Flights	Skewness	Global Moran’s I Z-Score
A	32	1	1.41	1.36
B	17	1	14.35	3.23
C	25	1	7.46	8.67
D	20	1	7.01	2.44

**Table 2:** Summary of the field test flights in the study area.

	Test	AGL Flight Height	Remediation Phase	Well Coverage
Survey 1	Flight E	25 m	Pre-Remediation	1, 2, 3
	Subset E-1	25 m	Pre-Remediation	1
	Subset E-2	25 m	Pre-Remediation	2, 3
	Flight F	20 m	Pre-Remediation	1
Survey 2	Flight G	25 m	Pre-Remediation	1, 2, 3
	Subset G-1	25 m	Pre-Remediation	1
	Subset G-2	25 m	Pre-Remediation	2, 3
	Flight H	20 m	Pre-Remediation	1
Survey 3	Flight I	25 m	During	1, 2, 3
	Flight J	20 m	During	1, 2, 3

## Data Availability

The data presented in this article are available on request from the corresponding author.
